# Editorial: The physiological relationship between sleep and exercise

**DOI:** 10.3389/fphys.2024.1419268

**Published:** 2024-05-08

**Authors:** Michele Lastella, Khaled Trabelsi, Mohamed Romdhani

**Affiliations:** ^1^ Appleton Institute for Behavioural Science, Central Queensland University, Rockhampton, QLD, Australia; ^2^ High Institute of Sports and Physical Education, University of Sfax, Sfax, Tunisia; ^3^ Physical Activity, Sport and Health, UR18JS01, National Observatory of Sports, Tunis, Tunisia; ^4^ Interdisciplinary Laboratory in Neurosciences, Physiology and Psychology: Physical Activity, Health and Learning (LINP2), UFR STAPS (Faculty of Sport Sciences), Paris Nanterre University, Nanterre, France

**Keywords:** physical activity, REM, polysomnography, actigraphy, recovery, training, competition, wellbeing

## Introduction

The interrelationship between sleep and exercise is a bidirectional one. It is well-established that sleep affects daytime functioning and physical exercise. Similarly, a plethora of evidence suggests that physical exercise can affect sleep quality and duration. To gain a comprehensive understanding of the physiological relationship between sleep and exercise, our Research Topic invited a wide variety of article types including original research, reviews, mini-reviews, and perspectives.

## Relationship between evening exercise and sleep

Theoretically, when exercise is performed too close to bedtime, the increase in core body temperature and/or the release of cortisol may potentially disrupt sleep drive. Miller et al. examined if sleep, exercise, and individual characteristics may be used to predict whether evening exercise will compromise subsequent sleep. The study was able to develop models to predict a high percentage of nights with compromised sleep based on individuals exercise and habitual sleep characteristics such that the algorithms indicate that evening exercise may only compromise a small percentage of nights (15% in females and 21% in males). Further, Perrier et al. examined the effects of both evening resistance or endurance exercises on sleep using polysomnography and salivary cortisol. They showed that endurance exercise led to a greater beta power during stage 2 sleep and rapid eye movement (REM) sleep as well as higher cortisol levels compared to the control condition. Comparable to an earlier report (Miller et al., 2020), these data indicate that while some changes were observed following evening endurance and resistance exercise, these changes were not harmful to the participants’ sleep.

## Napping

Athletes are exposed to several stressors that impair sleep, including training load, competition demands, early morning training, travel, and altitude exposure. As a result, napping has been used as a strategic tool to offset the adverse short- and long-term impacts of inadequate sleep. While the benefits of napping has been well-documented (Lastella et al., 2021), data is scarce when examining napping in conjunction with subsequent motivation music to further improve performance. Bentouati et al. observed that the combination of having a 30-min nap and listening to self-selected motivational music during warm-up (compared to control, napping alone, or listening to music alone) could be an effective strategy to enhance cognitive and physical performance decline induced by an exhaustive physical exercise.

## Balneotherapy, exercise and sleep

Castelli et al. conducted a systematic review examining whether balneotherapy can improve sleep quality in concomitance or not with exercise. Their findings revealed the complexity of examining balneotherapy and subsequent sleep behaviours due to the variability of treatments used across the twenty-one studies examined. However, 60% of the studies using balneotherapy only, and 88% combining balneotherapy and exercise improved self-perceived sleep quality, and at least 4 weeks of therapy are necessary to implement long-lasting sleep enhancement. The authors suggested that sleep enhancement may be attributed to the relaxing effect of balneotherapy and exercise (e.g., endorphin release, core body temperature regulation, pain threshold increase, and stress and anxiety decrease).

## Sleep, hematologic profile, and aerobic and anerobic capacity

Kraemer et al. aimed to apply a complex network model to verify the influence of sleep and hematological variables on aerobic and anaerobic work capacity. The complex network model provided insights into the link between sleep and hematologic profile such that total sleep duration was associated with basophils count (a white blood cell subgroup that are activated during chronic inflammation, infections, and allergic reactions), which in turn (i.e., basophil count), appeared to be influential toward aerobic and anaerobic capacities. Further, higher daytime sleepiness indirectly influenced anaerobic work capacity through higher platelet count and mean platelet volume.

## Lockdown duration and training intensity on sleep

Given the impact COVID-19 had on athlete’s ability to train and compete, Romdhani et al. conducted a large international (40 countries) retrospective, cross-sectional survey exploring 1,454 elite athletes sleep and training behaviours pre- and during COVID-19 lockdown. While the total sleep duration and time spent in bed increased during lockdown, athletes reported reduced sleep quality and increased insomnia. The proportion of athletes reporting moderate insomnia increased three-fold and severe insomnia six-fold, during lockdown. The authors showed that athletes who maintained (compared to those who did not) high training intensity reported lower levels of insomnia and overall better sleep quality during lockdown. Further, athletes spending more than 2 months in lockdown reported higher insomnia severity compared to athletes spending less than a month in lockdown. The relevance of these data are not only significant for future lockdowns but, also, for situations such as illness, injury, and quarantine after international travel.

## Athlete retirement and sleep

Montero et al. examined what factors were associated with symptoms of sleep and mental health disorders in retired athletes. Following a large scale survey investigating 173 former athletes (50% women), findings revealed that age and gender were associated with symptoms of anxiety. Women exhibited higher likelihood of experiencing anxiety symptoms, while each year increase in age was associated with a 5% decrease in likelihood of experiencing anxiety. Further analyses revealed increased body mass was related with an elevated risk for sleep difficulty, sleep disordered breathing, and compromised wellbeing. The links between higher body mass and adverse health outcomes are thought to stem from deregulation of eating behaviours and reduced physical activity levels following retirement (Witkowski and Spangenburg, 2008; Yao et al., 2020).

## Overview

Taken together, the articles included in the current Research Topic highlight the interaction between sleep and physical activity, with the two limbs of the equation reciprocally affecting each other. Clearly, both short-term (during the lockdown, Romdhani et al.) and long-term (after retirement from sport, Montero et al.) reduced physical activity (intensity and/or volume) contribute to lower sleep quality, while increased physical activity level contribute to an enhanced subjective perception of sleep (Castelli et al.). On the other hand, sleep duration and daytime sleepiness indirectly influence (through higher basophil and platelet count) aerobic and anaerobic performances (Kreamer et al.). Even short sleep bouts (notably when combined with listening to self-selected motivational music during the warm-up) could have potential ergogenic effects on physical and cognitive performances and perceived effort (Bentouati et al.). Not only evening exercise would affect the architecture of the subsequent sleep, the nature of the exercise itself (e.g., endurance or resistance exercise) would affect sleep architecture differently (Perrier et al.). It is noteworthy to mention that evening exercise might be more disturbing for males than females (Miller et al.).

## Conclusion

The current Research Topic furthers the existing knowledge about the bidirectional relationship between sleep and physical exercise. Indeed, the intensity, volume, timing, and even the nature of the exercise may affect sleep quantity, quality, and architecture. Additionally, shorter sleep duration and higher daytime sleepiness may reduce aerobic and anaerobic capacities, while napping seems to be protective against the exercise-induced exertion.
